# A single-session assessment: an opportunity for psychoeducation as intervention

**DOI:** 10.1186/2050-2974-3-S1-O14

**Published:** 2015-11-23

**Authors:** Sharon Ridley, Susan Byrne, Anthea Fursland, David Erceg-Hurn, Peter McEvoy

**Affiliations:** 1 Centre for Clinical Interventions, Australia; 2 University of Western Australia, Australia; 3 Curtin University of Technology, Australia

## 

With limited resources, public clinics tend to have long waiting lists. At the Centre for Clinical Interventions Eating Disorder Programme a ~6-month waiting list has been the norm. In May 2014, with 70 patients and a 9 month wait for treatment, we introduced a new assessment process intended to improve efficiency. Within two weeks of being referred, patients are offered a “Single-session Assessment Appointment” (A0) with a senior clinical psychologist to determine their suitability for the programme. Patients complete questionnaires (demographic and psychometric information), receive a clinical interview, and have their height and weight measured. They receive verbally and in writing: a) psycho-education; b) an orientation to Enhanced Cognitive Behaviour Therapy; and c) alternative services and emergency contacts. Patients provide consent to remain on the waiting list for treatment, and receive a further assessment (A1) immediately prior to commencing treatment. Preliminary findings of A0 to A1 changes indicate a reduction of almost 50% on both waitlist and waiting time, and >50% of patients making positive changes (e.g., reduction or cessation of purging). Early results from this new assessment process suggest the potential impact of psychoeducation as early intervention, allowing changes to begin at A0, months before the start of treatment.

